# Structural and microvascular retinal changes following bariatric surgery: a prospective study

**DOI:** 10.1186/s40942-026-00819-0

**Published:** 2026-02-26

**Authors:** Lívia da Silva Conci, Leandro Bortolon Bissoli, Davi Paraguassu de Sousa Martins, Cleide Guimarães Machado, André Carvalho Kreuz, Rony Carlos Preti, Leonardo Proveti Cunha, Alexandre Grobberio Pinheiro, Marco Aurelio Santo, Mario Luiz Ribeiro Monteiro, Leandro Cabral Zacharias

**Affiliations:** 1 Department of Ophthalmology, University of São Paulo, 255 Dr. Enéas de Carvalho Aguiar Avenue, São Paulo, SP 05403000 Brazil; 2 Department of Ophthalmology, Federal University of Juiz de Fora, Minas Gerais, Brazil; 3 Department of Ophthalmology, Federal University of Espírito Santo, Espírito Santo, Brazil; 4 Department of Gastroenterology, University of São Paulo, São Paulo, Brazil

**Keywords:** Obesity, Diabetes, Retina, Diabetic retinopathy, Bariatric surgery, Vascular density, Optical coherence tomography (OCT), Optical coherence tomography angiography (OCTA)

## Abstract

**Background:**

This study sought to evaluate structural retinal changes, mainly microvascular ones, in obese patients undergoing bariatric surgery.

**Methods:**

Obese patients who were candidates for bariatric surgery at University of São Paulo Medical School Clinics Hospital were recruited. Each participant underwent ophthalmic evaluation with ultra-widefield retinography, optical coherence tomography (OCT) and optical coherence tomography angiography (OCTA) obtained within one month before and six months following the surgical procedure. Structural OCT parameters (subfoveal choroidal thickness, mean peripapillary nerve fiber layer thickness, central foveal thickness, and macular volume) and OCTA parameters (peripapillary vascular density, macular vascular density, area, perimeter and circularity of the foveal avascular zone - FAZ - of both superficial and deep vascular complexes) were calculated and compared.

**Results:**

42 participants were included (83.3% female, mean age 47 years) of whom 24 (57.14%) were diabetic and 18 (42.86%) were non-diabetic. Of this sample, 81 eyes were evaluated. Six months after bariatric surgery, a significant increase in central foveal thickness (from 258±26 μm to 260±25 μm, *p* < 0.001), macular volume (from 8.6 ± 0.4 mm³ to 8.7 ± 0.4 mm³, *p* < 0.001), peripapillary vascular density (from 63 ± 8% to 65 ± 8%, *p* = 0.003) and macular vascular density in the superficial (from 27 ± 4.7% to 28 ± 4.4%, *p* = 0.005) and deep vascular complexes (29 ± 3.9% to 31 ± 3.5%, *p* < 0.001) were observed. No significant changes were detected in the metrics of the FAZ, subfoveal choroidal thickness and mean peripapillary nerve fiber layer thickness.

**Conclusions:**

In this study, bariatric surgery was associated with improved retinal microvascular perfusion, as demonstrated by the increase in macular and peripapillary vascular density. The increase in central foveal thickness and macular volume may be secondary to improved microvascular perfusion.

## Background

Obesity is a highly prevalent global condition, which may affect several systemic functions. It is defined as a body mass index (BMI) greater than 30 kg/m², and it is characterized by a complex and multifactorial pathophysiology [1]. Obesity is strongly related to an increased risk of developing diabetes mellitus (DM) and cardiovascular diseases [2].

Treatment options for obesity may include lifestyle changes, pharmacological therapies, and surgical procedures. Among these, bariatric surgery has shown superior long-term outcomes in achieving glycemic control and DM remission, when compared to medical therapy [3]. It is well known that glycemic control is well established as a protective factor against the onset and the progression of diabetic retinopathy (DR). However, abrupt and intensive control of glucose levels may paradoxically induce worsening of DR, whose pathogenesis has not been fully elucidated yet [4]. The evaluation of retinal microcirculation can provide valuable insights into the early detection, prognosis, and monitoring of several chronic metabolic diseases [5]. With the recent advent of optical coherence tomography angiography (OCTA), it has become possible to noninvasively assess microvascular alterations in vivo.

The literature reports conflicting findings, with positive, negative, and even neutral effects of bariatric surgery on the progression of DR [6]. In most studies, DR analysis was based primarily on clinical findings from fundus examination and posterior pole photography, so few publications have sought to analyze subclinical changes using OCTA [7–10]. Considering the growing number of obese individuals undergoing bariatric surgery, the purpose of this study was to prospectively evaluate structural retinal changes, especially the microvascular ones, in obese patients undergoing bariatric surgery.

## Methods

### Study design and population

This prospective study was conducted at the University of Sao Paulo Medical School Clinics Hospital (HCFMUSP), located in São Paulo, Brazil. It was conducted in accordance with the Declaration of Helsinki and it was approved by the HCFMUSP Research Ethics Committee, under registration number 29546120.2.0000.0068. Written informed consent was obtained from all participants.

Obese patients eligible for primary bariatric surgery at HCFMUSP were recruited. Additional inclusion criteria were appropriate endocrinological follow-up (for those diagnosed with DM), age between 18 and 70 years, and ability to understand the study procedures. Exclusion criteria comprised (1) systemic diseases potentially affecting the retina (such as sickle cell disease, neoplastic disorders, or rheumatologic conditions), (2) ocular conditions compromising image quality or retinal assessment (e.g. media opacities, glaucoma, macular degeneration, spherical equivalent greater than ± 5 diopters, indication for or history of panretinal laser photocoagulation, vitreoretinal interface disorders, such as vitreomacular traction syndrome or epiretinal membrane grades 2–4 according to Govetto et al. [11]), (3) prior ophthalmic surgery (except for uncomplicated phacoemulsification performed over three months prior to enrollment), and (4) inability to cooperate with exams.

Data collection was conducted between March 2021 and December 2023. Anthropometric measurements, including weight and BMI, besides fasting blood sugar and glycated hemoglobin (HbA1c) measurements were performed at the bariatric unit of the hospital. All subjects underwent a comprehensive ocular examination, including best-corrected visual acuity (BCVA), refraction and intraocular pressure measurement by Goldmann applanation tonometer. Anterior and posterior segment examinations were performed to rule out any ocular disorder. Each participant underwent ultra-widefield retinography using a nonconfocal ultra-widefield scanning laser ophthalmoscope (SLO) (Daytona, Optos, Dunfermline, UK), as well as optical coherence tomography (OCT) and OCTA, obtained up to one month before (T0) and six months following the surgical procedure (T6).

### OCT and OCTA imaging protocol

Spectral-domain OCT and OCTA images were acquired using the SPECTRALIS^®^ OCT module (Heidelberg Engineering, GmbH, Heidelberg, Germany). The macular and peripapillary OCT scanning protocols were used, with repeated acquisitions of each scan type with Automatic Real-time Tracking (ART), and an acquisition quality index of at least 25. Images with significant artifacts due to motion, projection, duplicated vessels, or distortions were reacquired, and all scans were manually reviewed to ensure adequate segmentation. Using the Enhanced Depth Image (EDI) tool, manual measurement of subfoveal choroidal thickness was possible.

The OCTA protocol consisted of obtaining 10°x10° images (512 A-scans/B-scans and 512 B-scans/volume) centered at the fovea and 15°x15° images (512 A-scans/B-scans and 512 B-scans/volume) centered at the optic disc. The axial resolution was 3.9 μm/pixel and the transverse resolution was 5.7 μm/pixel. En face OCTA images of the superficial vascular complex (SVC) and deep vascular complex (DVC) were generated using automatic segmentation of the retinal layers by Spectralis software. The superior and inferior limits of the SVC were, respectively, the internal limiting membrane and 17 μm above the inferior border of the inner plexiform layer. The superior and inferior limits of the DVC were defined, respectively, as 17 μm above the inferior border of the inner plexiform layer and the outermost portion of the outer plexiform layer. Low-quality scans were excluded from the analysis if any of the following criteria were met: (1) images with impaired sharpness; (2) low signal intensity caused by artifacts; (3) motion artifacts, visible as irregular vessel patterns; and (4) images with a decentralized fovea.

The quantitative evaluation of macular and peripapillary OCTA images was based on the publication by Mello et al. [12], in which vascular densities and foveal avascular zone (FAZ) parameters area (mm²), perimeter (mm), and circularity of both superficial and deep vascular complexes were calculated using ImageJ software (National Institutes of Health, Bethesda, Maryland, USA; available at https://imagej.nih.gov/ij/download.html). OCTA reports were exported in TIFF format and cropped (macular: 962 × 962 pixels, fovea-centered; peripapillary: 938 × 938 pixels, optic disc–centered). Images were binarized using a global Otsu threshold, with white pixels representing vessels. Vascular density (%) was calculated as the ratio between vessel area and total area. FAZ segmentation of superficial and deep vascular complexes was automatically performed using the Level Sets algorithm, generating area, perimeter, and circularity metrics. For peripapillary analysis, a 1.7–3.4 mm annular region centered on the optic nerve head was evaluated globally and by standard sectors.

Therefore, structural OCT parameters (subfoveal choroidal thickness, mean peripapillary nerve fiber layer thickness, central foveal thickness, and macular volume) and OCTA parameters (peripapillary vascular density, macular vascular density, area, perimeter and circularity of FAZ - of both superficial and deep vascular complexes) were calculated and compared within one month before and six months after bariatric surgery. Differences between patients with and without DM, with and without hypertension, and type of bariatric surgery (sleeve gastrectomy or Roux-en-Y gastric bypass - RYGB) were also assessed.

### Statistical analysis

Statistical analysis was performed by an independent statistician using R software (version 4.4.2; R Foundation for Statistical Computing, Vienna, Austria). Descriptive statistics were calculated and the data were summarized as mean, standard deviation (SD), minimum, median, and maximum values for quantitative variables. Categorical variables were described as absolute and relative frequencies. Longitudinal analyses were conducted using Generalized Linear Mixed Models (GLMM). To assess differences over time between groups (with and without DM, with and without hypertension, and between types of bariatric surgery), Mann–Whitney tests were applied to continuous variables. Correlations between variables were assessed using Spearman’s correlation coeficient. The results were considered statistically significant with *p* ≤ 0.05.

## Results

### Baseline clinical and demographic characteristics

A total of 42 subjects were included (83.3% female, mean age 47 years) of whom 24 (57.14%) were diabetic and 18 (42.86%) were non-diabetic. Systemic hypertension was observed in 28 (66.7%) participants. Of this sample, 81 eyes were evaluated. In 3 patients (7%), only one eye was evaluated, due to unilateral findings of corneal opacity (1), refractive amblyopia (1) and macular scar from retinochoroiditis (1). The main clinical and demographic characteristics of study population are detailed in Table 1.

**Table 1:** Baseline demographic and clinical characteristics of the study population

Variable	Global sample (42 subjects)
Age (years) (mean ± SD*)	47.4 ± 10.9
Number of eyes included from each participant:	
1 (N, %)	3 (7.1%)
2 (N, %)	39 (92.9%)
Sex:	
Female (N, %)	35 (83.3%)
Male (N, %)	7 (16.7%)
Ethnicity:	
Asian (N, %)	1 (2.38%)
Caucasian (N, %)	29 (69.0%)
Black (N, %)	2 (4.76%)
Mixed-race (N, %)	10 (23.8%)
Diabetes Mellitus:	
No (N, %)	18 (42.9%)
Yes (N, %)	24 (57.1%)
Systemic Arterial Hypertension:	
No (N, %)	14 (33.3%)
Yes (N, %)	28 (66.7%)
Dyslipidemia:	
No (N, %) Yes (N, %)	27 (64.3%) 15 (35.7%)
Coronary artery disease:	
No (N, %) Yes (N, %)	40 (95.2%) 2 (4.76%)
Smoking:	
No (N, %) Yes (N, %)	35 (83.3%) 7 (16.7%)
Bariatric Surgery Technique	
Roux-en-Y gastric bypass (N, %) Vertical gastrectomy (sleeve) (N, %)	31 (73.8%) 11 (26.2%)

* SD: standard deviation

Among the 47 eyes from 24 participants with diabetes, 37 eyes (78.72%) showed no clinically detectable diabetic retinopathy at baseline, while 10 eyes (21.27%) had diabetic retinopathy (6 mild and 4 moderate nonproliferative DR). At 6 months, ultra-widefield imaging showed no new cases of DR and limited progression in two eyes, from mild to moderate nonproliferative DR due to the appearance of a single microhemorrhage.

### Laboratory and anthropometric outcomes

Laboratory and anthropometric data showed statistically significant reductions during follow-up (*p* < 0.001). The mean fasting glucose decreased from 129.0 mg/dL (SD = 45.7) to 92.0 mg/dL (SD = 24.2), while HbA1c declined from 7.1% (SD = 2.0) to 5.4% (SD = 0.7). The mean BMI decreased from 47.0 to 36.9 kg/m², and the mean body weight decreased from 125.0 kg to 97.6 kg. The data are summarized in Table 2.

**Table 2:** Comparison of laboratory and anthropometric parameters before and 6 months after surgery

Variable	Time	*N*	Mean	SD	Minimal	Median	Maximum	*p*-value
Fasting blood glucose (mg/dL)	T0	42	129.0	45.7	72.0	117.0	253.0	**< 0.001**
	T6	42	92.0	24.2	62.0	84.5	187.0	
HbA1c (%)	T0	42	7.1	2.0	5.2	6.4	13.5	**< 0.001**
	T6	42	5.4	0.7	4.0	5.2	7.2	
BMI (kg/m²)	T0	42	47.0	7.6	32.6	48.6	66.0	**< 0.001**
	T6	42	36.9	7.4	25.3	35.3	55.9	
Weight (kg)	T0	42	125.0	26.0	78.4	126.0	189.0	**< 0.001**
	T6	42	97.6	22.0	60.0	96.5	139.0	

SD: standard deviation; T0: before bariatric surgery; T6: six months after bariatric surgery; HbA1c: glycated hemoglobin; BMI: body mass index

### OCT and OCTA quantitative parameters

**Fig. 1:**
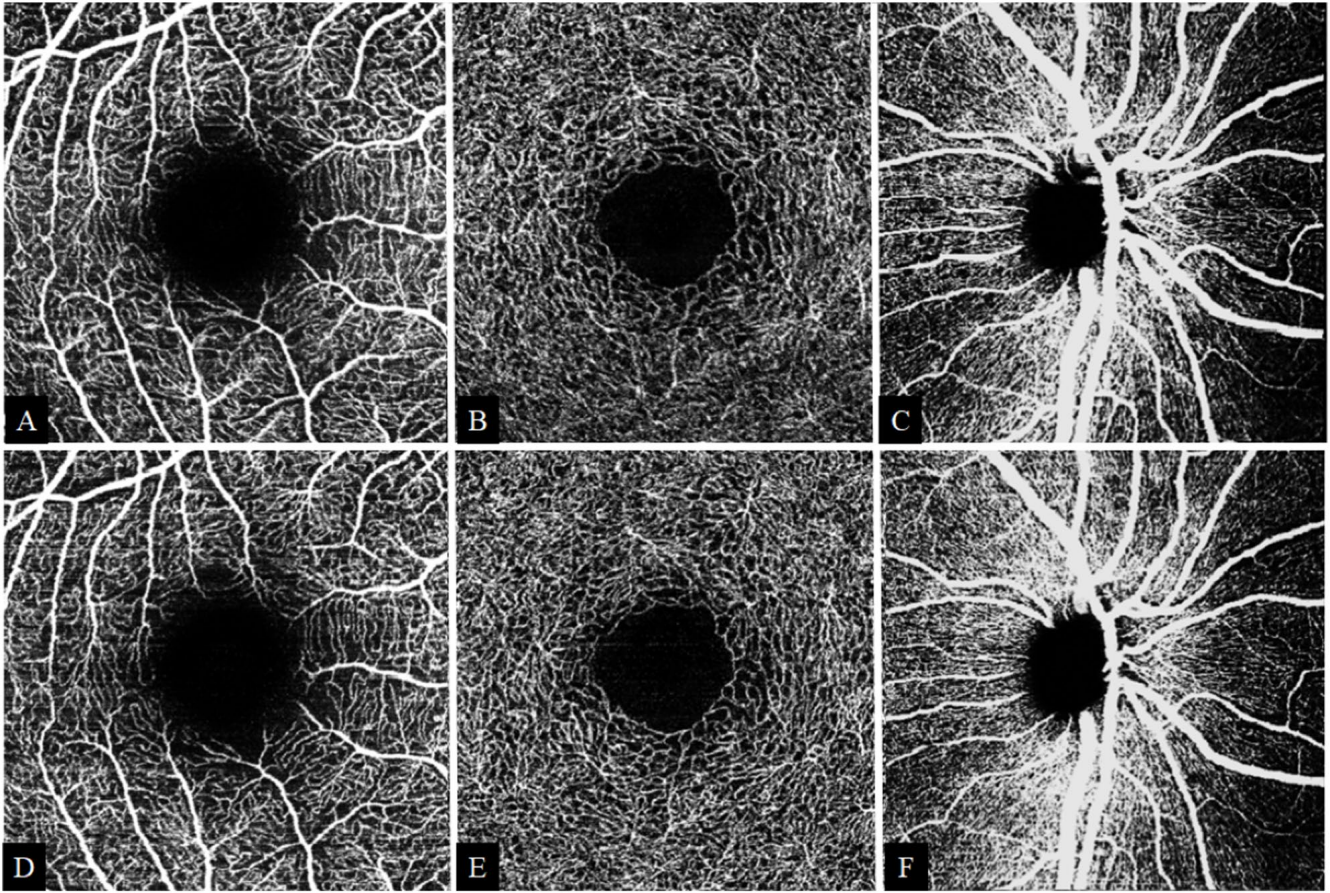
Representative *en face* Spectralis^®^ OCTA images from a study participant (right eye). Images in the upper row (A–C) were acquired preoperatively and those in the lower row (D–F) postoperatively. A and D show the macular superficial vascular complex (10° × 10°); B and E, the macular deep vascular complex (10° × 10°); and C and F, the peripapillary superficial vascular complex (15° × 15°), which includes large vessels. Images are raw en face OCTA exports and scans from both time points show matched localization and comparable image quality

At six months postoperatively, significant increases in central foveal thickness (from 258±26 μm to 260±25 μm, *p* < 0.001), macular volume (from 8.6 ± 0.4 mm³ to 8.7 ± 0.4 mm³, *p* < 0.001), peripapillary vascular density (from 63 ± 8% to 65 ± 8%, *p* = 0.003) and macular vascular density in the superficial (from 27 ± 4.7% to 28 ± 4.4%, *p* = 0.005) and in the deep vascular complexes (29 ± 3.9% to 31 ± 3.5%, *p* < 0.001) were observed (Table 3). No significant changes were detected in the FAZ metrics, subfoveal choroidal thickness, or mean peripapillary nerve fiber layer thickness. Significant increases in peripapillary SVC (pSVC) vascular density were observed for the overall mean, as well as in the inferior, temporal, and nasal sectors (Table 4). Table 5 presents the detailed sectoral changes in macular vascular density in the superficial and deep vascular complex. For illustrative purposes, representative preoperative and postoperative OCTA images are shown in Fig. 1.

**Table 3:** Comparison of quantitative parameters of OCT and OCTA before and 6 months after surgery

Variable	Time	*N*	Mean	SD	Minimal	Median	Maximum	*p*-value
Central foveal thickness (µm)	T0	81	258	26	221	255	347	**< 0.001**
	T6	81	260	25	221	259	348	
Macular volume (mm³)	T0	81	8.6	0.4	7.7	8.7	9.2	**<0.001**
	T6	81	8.7	0.4	7.9	8.7	9.5	
Choroidal thickness (µm)	T0	81	290	88	105	281	540	0.685
	T6	81	286	89	147	270	579	
Mean NFL Thickness (µm)	T0	78	101	11.1	75	99.5	124	0.265
	T6	78	100	10.9	76	100	122	
FAZ SVC area (x10-2 mm^²)^	T0	77	8.6	0.16	8.2	8.7	8.9	0.871
	T6	77	8.6	0.18	8.3	8.6	9.1	
FAZ DVC area (x10-2 mm^²^)	T0	77	8.7	0.14	8.4	8.7	9.0	0.798
	T6	77	8.7	0.13	8.4	8.7	8.9	
FAZ SVC perimeter (mm)	T0	77	12.0	0.03	11.9	12.0	12.1	0.246
	T6	77	12.0	0.05	11.9	12.0	12.1	
FAZ DVC perimeter (mm)	T0	77	12.0	0.03	12.0	12.0	12.1	0.655
	T6	77	12.0	0.04	12.0	12.0	12.1	
FAZ SVC circularity	T0	77	0.75	0.014	0.72	0.75	0.77	0.068
	T6	77	0.75	0.015	0.72	0.75	0.79	
FAZ DVC circularity	T0	77	0.75	0.011	0.73	0.76	0.78	0.478
	T6	77	0.75	0.011	0.73	0.75	0.77	
Macular SVC density (%)	T0	79	27	4.7	18	27	36	**0.005**
	T6	79	28	4.4	14	29	37	
Macular DVC density (%)	T0	79	29	3.9	18	29	37	**< 0.001**
	T6	79	31	3.5	21	31	40	

SD: standard deviation; T0: before bariatric surgery; T6: six months after bariatric surgery; NFL: nerve fiber layer; FAZ: foveal avascular zone, SVC superficial vascular complex; DVC: deep vascular complex

**Table 4:** Comparison of vascular density in peripapillary superficial vascular complex before and 6 months after surgery

Variable (VD)	Time	*N*	Mean	SD	Minimal	Median	Maximum	*p*-value
Overall pSVC (%)	T0	78	63	8	38	65	76	**0.003**
	T6	78	65	8	46	66	77	
Superior pSVC (%)	T0	78	76	11	47	78	91	0.560
	T6	78	76	11	39	78	91	
Inferior pSVC (%)	T0	78	80	9	45	82	92	**0.001**
	T6	78	82	8	54	84	93	
Temporal pSVC (%)	T0	78	46	13	11	45	68	**< 0.001**
	T6	78	50	12	20	51	79	
Nasal pSVC (%)	T0	78	56	12	26	55	78	**0.046**
	T6	78	57	13	30	59	81	

SD: standard deviation; T0: before bariatric surgery; T6: six months after bariatric surgery; pSVC: peripapillary superficial vascular complex; VD: vascular density

**Table 5:** Comparison of sectorial macular vascular density in both superficial and deep vascular complex before and 6 months after surgery

Variable (VD)	Time	*N*	Mean	SD	Minimal	Median	Maximum	*p*-value
Superior SVC (%)	T0	77	27.3	5.6	14.0	27.3	39.9	**0.005**
	T6	77	28.9	5.6	13.3	29.5	40.0	
Superior DVC (%)	T0	77	28.9	4.9	16.1	28.9	38.3	**0.032**
	T6	77	30.2	5.1	17.2	31.0	40.8	
Inferior SVC (%)	T0	77	27.7	5.6	15.8	27.7	37.0	0.069
	T6	77	28.8	5.5	12.1	28.6	41.7	
Inferior DVC (%)	T0	77	26.3	5.4	11.5	27.1	37.2	**< 0.001**
	T6	77	28.7	4.7	15.1	28.6	38.7	
Nasal SVC (%)	T0	77	23.6	5.2	12.9	24.0	37.4	0.077
	T6	77	24.7	4.5	9.8	25.2	32.3	
Nasal DVC (%)	T0	77	29.6	4.8	20.1	29.7	43.0	**0.002**
	T6	77	31.3	4.4	20.0	31.3	42.2	
Temporal SVC (%)	T0	77	28.6	6.6	12.4	28.7	41.1	**0.040**
	T6	77	30.1	5.5	17.9	30.5	40.1	
Temporal DVC (%)	T0	77	30.4	5.9	14.3	31.9	40.1	**0.001**
	T6	77	32.3	4.4	19.8	33.4	41.9	

SD: standard deviation; T0: before bariatric surgery; T6: six months after bariatric surgery; SVC superficial vascular complex; DVC: deep vascular complex; VD: vascular density

No statistically significant difference over time was observed between the groups with and without DM, arterial hypertension, and according to the bariatric surgery technique.

### Correlation analysis of outcomes and clinical data

Table 6 presents the Spearman correlations between changes in OCT and OCTA parameters and variations in BMI, fasting blood glucose, and HbA1c. Significant negative correlations were found:

- Reduction in BMI was correlated with an increase in overall macular VD;
- Fasting glucose reduction was associated with increased macular volume, choroidal thickness, and mean RNFL thickness;
- HbA1c reduction correlated with increased central foveal thickness, macular volume, choroidal thickness, and mean RNFL thickness.

**Table 6:** Spearman’s correlation coefficient between changes in OCT and OCTA parameters and variations in BMI, fasting blood glucose and HbA1c

Parameter	BMI	Fasting blood glucose	HbA1c
Central Foveal Thickness (µm)	-0.02	-0.11	**-0.25**
Macular Volume (mm³)	0.09	**-0.23**	**-0.25**
Choroidal Thickness (µm)	0.02	**-0.28**	**-0.27**
Mean Thickness NFL (µm)	0.04	**-0.30**	**-0.22**
FAZ SVC Area (x10-2 mm^²^)	0.12	-0.04	0.09
FAZ DVC Area (x10-2 mm^²)^	0.05	-0.22	0.03
FAZ SVC Perimeter (mm)	0.04	-0.05	0.06
FAZ DVC Perimeter (mm)	-0.03	-0.18	0.09
FAZ SVC Circularity	0.12	-0.03	0.05
FAZ DVC Circularity	0.07	-0.13	-0.13
Overall SVC Density (%)	0.05	0.00	0.00
Overall DVC Density (%)	**-0.33**	0.21	-0.05
Superior SVC Density (%)	0.07	-0.07	-0.02
Superior DVC Density (%)	-0.22	0.10	-0.06
Inferior SVC Density (%)	-0.03	0.07	-0.08
Inferior DVC Density (%)	-0.17	0.19	0.03
Nasal SVC Density (%)	0.09	0.02	0.02
Nasal DVC Density (%)	-0.14	0.01	-0.11
Temporal SVC Density (%)	-0.01	-0.05	-0.02
Temporal DVC Density (%)	-0.19	0.18	-0.01
Overall pSVC Density (%)	0.05	0.04	-0.09
Superior pSVC Density (%)	0.03	0.01	-0.11
Inferior pSVC Density (%)	-0.03	0.03	-0.02
Temporal pSVC Density (%)	0.00	-0.08	-0.01
Nasal pSVC Density (%)	0.08	0.07	-0.09

HbA1c: glycated hemoglobin; BMI: body mass index; NFL: nerve fiber layer; FAZ: foveal avascular zone, SVC superficial vascular complex; DVC: deep vascular complex; pSVC: peripapillary superficial vascular complex

## Discussion

In this study, 81 eyes from 42 obese subjects were investigated for the effect of bariatric surgery on structural retinal changes. We observed a significant increase in central foveal thickness and macular volume at 6 months, without the development of macular edema. These findings are consistent with several previous studies: Dogan et al. documented increased macular thickness in obese patients undergoing sleeve gastrectomy [13], while Posarelli et al. studied a sample of obese patients undergoing RYGB and found an increase in foveal thickness [14]. Brynskov et al. demonstrated macular thickening peaking at 6 months in patients with type 2 DM undergoing bariatric surgery. Similarly, retinal thickness correlated inversely with HbA1c levels [15]. Laiginhas et al. also documented significant thickening across macular regions, regardless of diabetes status, and attributed these changes to improved microvascular perfusion after surgery [16].

Recent studies indicate a strong correlation between retinal microvasculature function and changes in macrovascular systems in various diseases and cardiovascular risk factors. Regarding potential benefits of bariatric surgery on microcirculation, few studies evaluated this issue using OCTA. Laiginhas et al. demonstrated, over 6–12 months, an improvement in retinal microvascular perfusion with increased parafoveal vascular density of the DVC, increased circularity, and a reduction in the perimeter of the FAZ in patients undergoing RYGB. These findings were independent from the presence or absence of DM. However, the decrease in HbA1c was associated with increased vascular density in the DVC [7]. ElShazly demonstrated higher vascular density in the deep capillary plexus at 3 months of follow-up in a cohort of 40 patients without comorbidities undergoing RYGB [8]. ElShazly also evaluated the effect of bariatric surgery on peripapillary flow using OCTA in 60 obese patients 3 months postoperatively and, unlike our study, found no significant differences in peripapillary microcirculation [9]. Finally, Chen et al. demonstrated an increase in vascular density in the parafoveal and in the global, parafoveal, and perifoveal deep capillary plexus in subjects undergoing sleeve gastrectomy at 6 months of follow-up [10].

Our study confirmed signs of improvement in macular and peripapillary microvascular perfusion, through increased vascular density in the SVC, DVC, and pSVC. Unlike a previous study [7], we did not observe significant changes in FAZ parameters. We believe this finding may be related to a possible technical limitation of the method used for FAZ metrics calculation, as well as to intrinsic features of our sample, composed mostly of patients without DR and under appropriate endocrinological follow-up.

The significant changes found in our study were independent of the surgical technique used and the participants’ preoperative clinical diagnosis of diabetes or hypertension. Diabetic patients had already undergone adequate endocrinological follow-up, which may explain why, in this sample, increase in vascular density did not differ among diabetic or non-diabetic patients. Interestingly, postoperative decrease in BMI was associated with an increase in microvascular density in the DVC. This could be explained by the fact that this vascular complex has lower flow and lower partial oxygen pressure, which would make it more sensitive to the beneficial vasomotor changes resulting from weight loss.

The mechanisms underlying the benefits of bariatric surgery on the retinal microvasculature still need to be elucidated. We hypothesize that hemodynamic factors, such as decreased systemic blood pressure levels, and mechanical factors, such as reduced retro-orbital fat, may be involved. Improvements in hemodynamic parameters of the ophthalmic artery flow assessed by Doppler ultrasonography have already been demonstrated in subjects undergoing bariatric surgery [17]. In addition, hormonal factors, such as lower leptin levels and improved insulin sensitivity, may lead to a decrease in the inflammatory cytokines and vasoconstrictor agents, which could explain the improved retinal capillary perfusion. In combination, these factors may contribute to the observed increases in vascular density in both retinal vascular complexes.

Our study has some limitations. First, we acknowledge that the lack of detail regarding systemic blood pressure levels before and after bariatric surgery is a drawback of our work, even though no differences in vascular density were found between hypertensive and non-hypertensive subjects. Another limitation relates to the sample size and follow-up time, with few subjects exhibiting clinical signs of DR. Finally, OCTA is a relatively new tool with intrinsic limitations, and some of the variations in vascular densities can be attributed to device-to-visit variability and are not clinically significant [18]. In addition, axial length correction was not performed, as axial length measurements were not available for this cohort. We acknowledge that this may influence vascular density and FAZ-related metrics. Although all the potential limitations, to our knowledge, this is the first Brazilian study that used OCTA to evaluate the microvascular changes after bariatric surgery in obese patients. We confirmed previous findings that bariatric surgery improves microcirculation in the macular region and provided new evidence of improved peripapillary perfusion. Thus, improved retinal microvascular perfusion following bariatric surgery may reflect systemic microvascular benefits, consistent with evidence linking retinal and macrovascular function. These findings emphasize the systemic value of bariatric surgery, suggesting that perfusion improvements may occur in other microvascular beds, potentially contributing to broader clinical benefits in obese patients.

## Conclusions

In conclusion, this study confirmed previous findings that bariatric surgery improves microcirculation in the macular region and presented novel evidence of enhanced peripapillary perfusion in a Brazilian cohort.

## Data Availability

No datasets were generated or analysed during the current study.

## Abbreviations

ART: Automatic Real-time Tracking
BCVA: Best-corrected visual acuity
BMI: Body mass index
DVC: Deep vascular complex
DM: Diabetes mellitus
DR: Diabetic retinopathy
EDI: Enhanced Depth Image
FAZ: Foveal avascular zone
HbA1c: Glycated hemoglobin
HCFMUSP: University of Sao Paulo Medical School Clinics Hospital
NFL: Nerve fiber layer
OCT: optical coherence tomography
OCTA: Optical coherence tomography angiography
pSVC: Peripapillary superficial vascular complex
RYGB: Roux-en-Y gastric bypass
SD: Standard deviation
SVC: Superficial vascular complex
